# Characterization of the first mitochondrial genome of a little Corella (*Cacatua sanguinea*) and its phylogenetic implications

**DOI:** 10.1080/23802359.2019.1682481

**Published:** 2019-10-26

**Authors:** Subir Sarker, Saranika Talukder, Michelle Sutherland, Jade K. Forwood, Karla Helbig, Shane R. Raidal

**Affiliations:** a Department of Physiology, Anatomy and Microbiology, School of Life Sciences, La Trobe University, Melbourne, VIC, Australia;; b School of Animal and Veterinary Sciences, Faculty of Science, Charles Sturt University, Albury, New South Wales, Australia;; c School of Agriculture and Food, Faculty of Veterinary and Agricultural Sciences, The University of Melbourne, Victoria, Australia;; d The Unusual Pet Vets, Frankston, VIC, Australia;; e School of Biomedical Sciences, Faculty of Science, Charles Sturt University, Albury, New South Wales, Australia

**Keywords:** Avian mtDNA, mitogenome phylogeny, order Psittaciformes, *Cacatua sanguinea*

## Abstract

This study was designed to sequence the first complete mitochondrial genome from a little corella (*Cacatua sanguinea*). The mitogenome sequence was circular and 16,695 bp in length. In comparison to other available mitogenome sequences belongs to *Psittacidae* species, this mitogenome encoded a conserved structure consisting of 13 protein-coding genes (PCGs), two rRNA genes, 22 tRNA genes. The lengths of 12S and 16S ribosomal RNA were 975 bp and 1582 bp, respectively. The overall base composition of the mitogenome of *C. sanguinea* was dominated by higher AT (53.0%) than GC (47.0%) content. The complete mitogenome sequence determined in this study is useful for understanding the more profound evolutionary history and the conservation of *C. sanguinea.*

*Psittaciformes* is a large and diverse avian that order contains over 370 species placed within approximately 74 genera, most of which are distributed in the tropical parts of the Southern Hemisphere (Astuti et al. 2006; White et al. 2011). Although most parrot taxonomists believe the order *Psittaciformes* to be monophyletic, and some studies based on morphological, biochemical, chromosomal, and allozyme data have been conducted, the within-order phylogeny is still controversial (Astuti et al. 2006). Cockatoos comprise the distinctive family *Cacatuidae*, a major lineage of the order of parrots (*Psittaciformes*) that are distributed throughout the Australasian region. However, the evolutionary history of cockatoos is not well understood. There are currently 21 accepted cockatoo species noted for their variation in plumage, which differ from *Nestoridae* and *Psittacidae* in a number of characteristics. Cacatuids possess a moveable head-crest, are larger than most Nestorids and psittacids, and lack the Dyck feather texture that Nestorids and Psittacids have for bright blue and green plumage (Higgins 1999). Cockatoos are restricted to the Australasian region (excepting New Zealand), ranging from the Philippines and eastern Indonesian islands of Wallacea to New Guinea, the Solomon Islands and Australia (Cameron and Cunningham 2006). To evaluate the process of diversity and evolutionary relationships among the majority of *Cacatuidae* species, a molecular phylogeny was constructed using a partial mitochondrial and nuclear DNA sequence, with the addition of five complete mitochondrial genomes, and demonstrated that the Little Corella positioned phylogenetically between the Western Corella (*Cacatua pastinator*) and Goffin’s Cockatoo (*Cacatua goffini*) (White et al. 2011). However, we believe that the complete mitogenome could play a significant role to provide more clear evolutionary relationships, the divergence time of speciation, as well as influencing conservation and management decisions of species. Therefore, this study was designed for sequencing the complete mitogenome of *C. sanguinea,* which will further strengthen our understanding of the species diversity, host phylogeny and ecological diversity of the species.

A swab sample moistened in sterile phosphate-buffered saline, of the choana and cloaca was taken from a free-ranging Little Corella (*C. sanguinea*) originating from Nunawading, Victoria (Sample ID: #46, GPS location: Latitude: 37°49′2.46″S, Longitude: 145°10′37.2″E) and was used in this study. Animal sampling was obtained in accordance with approved guidelines set by the Wildlife and Small Institutions Ethics Committee (DEDJTR Victoria) under application number 01.15 (Sutherland et al. 2019) and the isolated genomic DNA was stored in −20 °C freezer at La Trobe University. The total genomic DNA was extracted using previously established protocol (Sarker, Das et al. 2018, 2017). The genomic library preparation and sequencing were performed according to the published protocol (Sarker, Roberts et al. 2017; Sarker, Isberg et al. 2018). Briefly, the paired-end library with an insert size of 301 bp was prepared using the Illumina Nextera XT DNA Library Perp V3 Kit (Illumina® Inc., San Diego, CA, USA) according to the manufacturer's instructions. Cluster generation and sequencing of the DNA-library were executed on Illumina® MiSeq chemistry according to the manufacturer’s instructions, which was generated approximately 10.98 million reads. The raw datasets were trimmed to pass the quality control based on PHRED score or per base sequence quality score, and the assembly of the mitochondrial genome was conducted using SPAdes assembler (version 3.10.0) (Bankevich et al. 2012) in Geneious. Annotation was performed using default parameter under Genetic code of vertebrate mitochondrial (transl_table 2) in Geneious (version 10.2.2).

The complete mitogenome sequence of *C. sanguinea* had a circular genome of 16,695 bp, containing 13 protein-coding genes (PCGs), 2 rRNA genes, and 22 tRNA genes. The contents of A, T, C, and G were 30.9, 22.1, 32.9, and 14.1, respectively. AT and GC contents of this complete mitogenome were 53.0 and 47.0%, respectively. The proportion of coding sequences with a total length of 11,278 bp (67.55%), which encodes 3748 amino acids, and all protein-coding genes started with Met. The lengths of 12S and 16S ribosomal RNA were 975 bp and 1582 bp, respectively. The gene arrangement was similar to the complete mitochondrial genome of other *Psittaciformes* species.

The complete mitogenome sequence of a *C. sanguinea* determined in this study, and other selected mitogenome sequences belong to the order *Psittaciformes* were retrieved from NCBI database and were aligned using the MAFFT L-INS-i algorithm in Geneious (Katoh et al. 2002). A maximum likelihood (ML) phylogenetic tree with 1000 non-parametric bootstrap resamplings was generated using Geneious (version 10.2.2). As highlighted in Figure 1, the mitogenome sequence of *C. sanguinea* produced a sub-clade with Indian ringneck parrot (*Psittacula krameri*; GenBank accession no. MN065674*)* (Sarker et al. 2019) and demonstrated a >91% pairwise nucleotide identity between them. We concluded that the complete mitogenome of *C. sanguinea* will be a useful database among the genus *Cacatua* to study the further host-phylogenetic relationship of *Cacatua* species, and suggests that this may be an implication for the conservation and management of the species.

**Figure 1.**
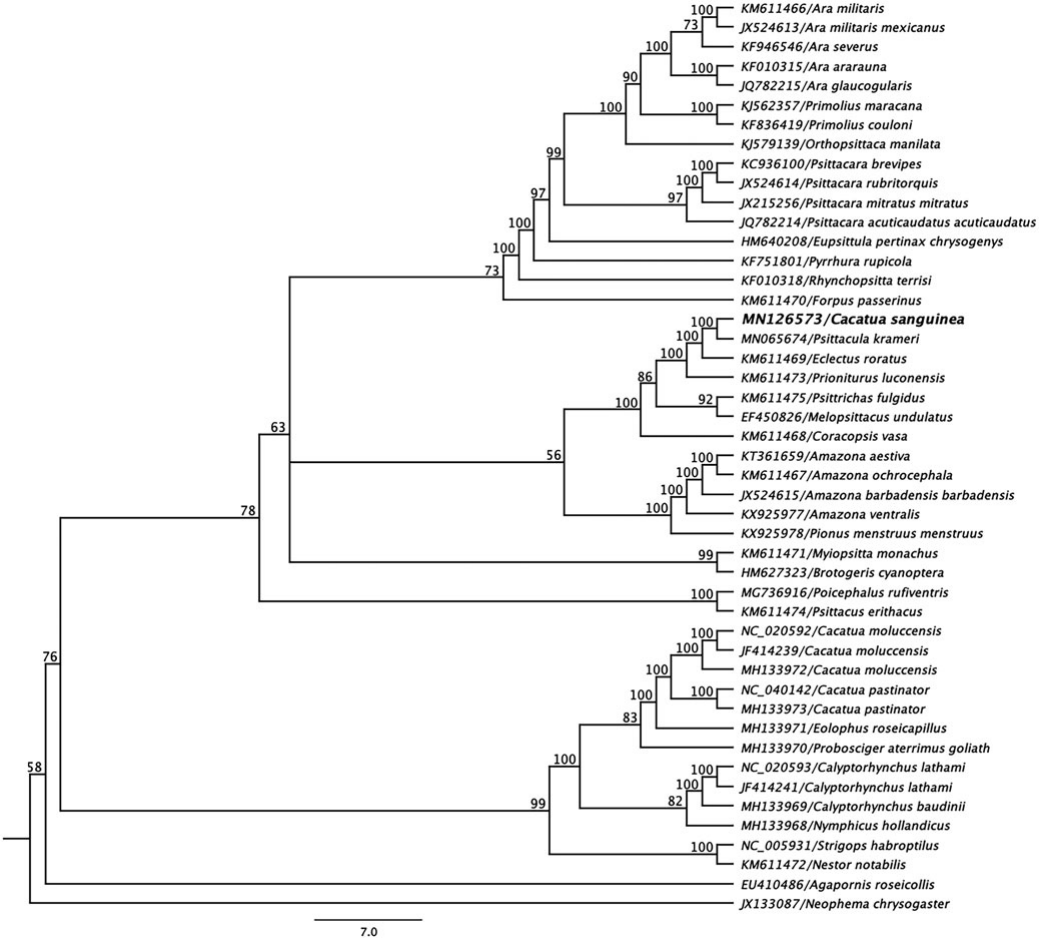
Maximum-likelihood phylogenetic tree to infer host-phylogeny relationship using mitochondrial genome sequenced from *C. sanguinea* along with others selected species under the order of *Psittaciformes*. The new complete mitogenome of *C. sanguinea* was highlighted by bold font.
